# Extended-Spectrum β-Lactamase and carbapenemase-producing *Escherichia coli* O157:H7 among diarrheic patients in Shashemene, Ethiopia

**DOI:** 10.1371/journal.pone.0306691

**Published:** 2024-08-01

**Authors:** Shimelis Teshome Ayalneh, Biruk Yeshitela Beshah, Yeonji Jeon, Seifegebriel Teshome, Tomas Getahun, Solomon Gebreselassie, Se Eun Park, Mekonnen Teferi, Woldaregay Erku Abegaz

**Affiliations:** 1 Department of Medical Laboratory Sciences, College of Health Sciences, Arsi University, Asela, Ethiopia; 2 Department of Microbiology, Immunology and Parasitology, School of Medicine, Collage of Health Sciences, Addis Ababa University, Addis Ababa, Ethiopia; 3 Bacterial and Viral Disease Research Directorate, Armauer Hansen Research Institute, Addis Ababa, Ethiopia; 4 Clinical, Assessment, Regulatory, Evaluation (CARE) Unit, International Vaccine Institute, Seoul, Republic of Korea; 5 Clinical Trials Directorate, Armauer Hansen Research Institute, Addis Ababa, Ethiopia; 6 Yonsei University Graduate School of Public Health, Seoul, Republic of Korea; Hawassa University College of Medicine and Health Sciences, ETHIOPIA

## Abstract

**Background:**

The worldwide increase in multidrug resistance is a major threat to public health. One particular concern is the presence of *Escherichia coli* strains that carry Extended-Spectrum β-Lactamase (ESBL) and Carbapenemase enzymes, which can make multiple antibiotics ineffective. This complicates treatment strategies and raises the risk of illness and death. The aim of this study was to isolate *E*. *coli* O157:H7, assess its susceptibility against antimicrobial agents, and determine the presence of ESBL and Carbapenemase production in stool samples collected from diarrheic patients in Shashemene, west Arsi, Ethiopia from July to November 2022.

**Methods:**

The samples were cultured McConkey Agar and *E*. *coli* were isolated and identified by standard biochemical tests using API 20E. *E*. *coli* O157:H7 was further identified using sorbitol McConkey Agar and antisera for O157 antigen test. The antimicrobial susceptibility test was performed using the Kirby–Bauer disc diffusion method using different antibiotics. Each identified isolate was screened and tested for phenotypical ESBL and Carbapenemase production using combined disc method and modified carbapenem inactivation method, respectively. Bivariant and multivariant analyses were employed using a logistic regression model for further analysis and were interpreted based on the odds ratio and level of statistical significance at a p-value <0.05 with 95% confidence interval.

**Results:**

*E*. *coli* O157:H7 strain was found from 9% (38/423) study participants. The majority of the participants [61.9% (262/423)] were males; and 19.1% (81/ 423) of the participants were under five children. Living in urban areas, having domestic animals, and ≥5 family size in the household were identified as statistically significant factors associated with *E*. *coli* O157:H7. Twenty-seven (71.1%) and 12 (31.6%) of the 38 *E*. *coli* O157:H7 isolates were phenotypically confirmed to be ESBL and carbapenemase producers, respectively. All isolates were resistant against Ampicillin, but sensitive to ciprofloxacin. High resistance to Ampicillin and Amoxicillin/Clavulanic acid was observed among the ESBL and carbapenemase producing isolates also. The extent of detection of multidrug resistant *E*. *coli* O157:H7 isolates against three or more classes of antimicrobial agents tested was alarmingly very high (84%).

**Conclusion:**

The *E*. *coli* O157:H7 isolates in this study showed a significant resistance to certain antimicrobials that were tested. The level of ESBL and Carbapenemase production among these isolates was found to be quite high. We observed a high resistance to Ampicillin and Amoxicillin/Clavulanic acid among the ESBL and carbapenemase producing isolates. Ciprofloxacin was found to be the most effective drug against both the ESBL producers and nonproducers.

## Introduction

Foodborne infections are a major cause of death and illness in low-income countries, resulting in significant healthcare costs [1]. *Escherichia coli* (*E*. *coli)* O157:H7, pose significant risks, with raw meat and dairy products being common sources [2–6]. While cattle are the main reservoir, other animals can also carry this pathogen [7–9].

Multidrug resistance has been increased all over the world, which is considered a public health threat. Several recent investigations reported the emergence of multidrug-resistant bacterial pathogens from different origins including humans, birds, cattle, and fish that increase the need for routine application of antimicrobial susceptibility testing to detect the antibiotic of choice and the screening of the emerging Multidrug resistance (MDR) strains [10, 11].

The emergence and global spread of antimicrobial resistance poses a significant threat to public health worldwide, especially in regions with limited resources and infrastructure for surveillance and control. Of particular concern are *E*. *coli* strains that carry Extended-Spectrum β-Lactamase (ESBL) and Carbapenemase enzymes, as they can render multiple antibiotics ineffective, complicating treatment strategies and increasing the risk of morbidity and mortality [12].

In the context of diarrheal diseases, the prevalence of ESBL and Carbapenemase-producing *E*. *coli* strains presents additional challenges in clinical management. These pathogens are resistant to commonly used antibiotics such as broad-spectrum β-lactams and carbapenems [13]. This situation is particularly alarming in low-income countries like Ethiopia, where diarrheal diseases are endemic and healthcare facilities may have limited resources to combat antimicrobial resistance [14, 15].

ESBL and Carbapenemase production by Shiga toxin-producing *E*. *coli* was formerly uncommon. However, recently, there are some reports of ESBL and carbapenems-producing *E*. *coli* O157:H7 from different parts of the world. The first report of ESBL-producing *E*. *coli* O157:H7 was reported in Netherland from chicken isolates in 2003 [16] and the first report from a human clinical sample isolate was reported in Denmark, which originated from a fecal sample of a 2-year-old girl with bloody diarrhea who was part of a small outbreak in 2004. Until this year, there was no report of such scenario from human clinical isolates [17]. There is also one study conducted in Japan that shows ESBL production among isolates found in beef cattle [18]. Another study from Egypt found that 63.0% of *E*. *coli* O157:H7 isolated from meat and dairy products carry β-Lactamase-encoding genes [19]. One study from Tanzania shows that 10% of isolate from cattle and 9.3% of isolate from human specimens have ESBL production capability [20].

To the best of our knowledge Even though there is little information regarding ESBL production in other parts of the world, there is no available published data regarding carbapenemase production of this bacteria in any part of the world. Regarding our country, it is similar to the world where no published data on ESBL and Carbapenamese production of this bacteria is available in Ethiopia.

The aim of this study was to investigate the prevalence, resistance to antimicrobials, and phenotypic characteristics of ESBL and Carbapenemase-producing *E*. *coli* O157:H7 strains among individuals suffering from diarrhea in Shashemene, Ethiopia. By acquiring a more comprehensive comprehension of the epidemiology and mechanisms of resistance within this specific context, valuable insights can be provided for local healthcare practices, initiatives on antimicrobial stewardship, and public health interventions that aim to control the dissemination of multidrug-resistant pathogens. Additionally, this research contributes to the global understanding of the dynamics of antimicrobial resistance and underscores the urgent requirement for coordinated strategies to address this escalating threat to human health.

## Method and materials

### Sample collection

This study was conducted in Shashemene Town and Shashemene Zuria Woreda under Ethiopian Cholera Control and Prevention (ECCP) project. The study site is selected because there were high number of diarrheal diseases reports from this area [21]. This study was a prospective healthcare facility-based (HCF-based) cross-sectional type that included 8 public healthcare facilities (HCFs) (Abosto, Awasho, Chebi, Toga, Harbate, Fajogole health centers as well as Melka oda and Shashemene hospitals) found in Shashemene. A total of 41,563 patients visited all the selected healthcare facilities, of whom 1,399 patients were screened for eligibility requirements; and 1,388 of them fulfilled the inclusion criteria. Among these patients, 423 were selected systematically and enrolled in the study. Stool Sample collections were done from July 2022 to November 2022. All collected samples were tagged by sample ID, date of sampling, and sample type and then transported to the Shashemane Specialized Hospital Microbiology laboratory for microbiological analysis using a cold chain.

### Bacterial isolation and identification

The stool sample were cultured and *E*. *coli* isolate were identified by colony characteristics on MacConkey agar (Condalab, Spain) as well as using Gram stain (Condalab, Spain), and API 20E (Biomerieux, France) standard biochemical tests. The isolates gave a distinct pink colony when growing on MacConkey agar due to ferementatio of lactose and colorless on Sorbitol MacConkey agar since this bacteria strain does not ferment sorbitol. Isolates also gave positive reaction for biochemical tests like Indole, catalase, lactose and glucose fermentation but negative for Urease production, Citrate utilization, hydrogen sulfide production, sorbitol fermentation and OxidaseFurther identification of *E*. *coli* O157:H7 was done using sorbitol utilization test on Sorbitol MacConkey agar (Condalab, Spain), and final serotyping was done using antisera for O157:H7 antigen (Denka, Japan).

#### Sample size calculation

The sample size required for this study is determined using a single proportions estimation method. In this method, the estimated proportion (P) is set at 50% because there hasn’t been a previous large-scale investigation of *E*. *coli* O157:H7 among diarrheic patients from the general population As a result, the total sample size required for the study is calculated to be 427.

### Antimicrobial susceptibility testing

A 0.5 MacFarland suspension of pure *E*. *coli* O157:H7 colonies was prepared in a normal saline solution. Inoculation of the test pathogen for the Antimicrobial susceptibility test (AST) tests was conducted on Muller-Hinton agar by soaking inoculating swab one time into a suspension and then swiping the overflown sample on the mouth of the tube using Lawn or carpet culture. Antimicrobials for sensitivity test were chosen based on CLSI recommendation for Enterobacteriaceae family. The AST was performed against the following antimicrobials which belong to 9 class (penicillin, beta-lactam, aminoglycoside, cephalosporin, quinolone, phenicol, macrolides, tetracycline, and carbapenem), (Ampicillin (10 μg), Amoxicillin-clavulanate (20/10 μg), Gentamicin (10 μg), Ceftriaxone (30 μg), Cefotaxime (30 μg), Ceftazidime (30 μg), Ciprofloxacin (5 μg), Trimethoprim-Sulfamethoxazole (1.25/23.75 μg), Chloramphenicol (30μg), Azithromycine (15 μg), Cefuroxime (30 μg), Tetracycline (30 μg), Imipenem(10μg), Ertapenem (10 μg), and Meropenem(10μg) (all produced by Condalab, Spain), using the Kirby–Bauer disc diffusion method, and the results were analyzed and interpreted according to CLSI guidelines [22]. Additionally, ESBL screening was conducted by assessing the zone of inhibition for the following antimicrobials: ceftazidime (30 μg), ceftriaxone (30 μg), and cefotaxime (30 μg). Screening for carbapenemase was also performed, using meropenem (10 μg), ertapenem (10 μg), or imipenem (10 μg).

### Extended Spectrum β-Lactamase production test

Phenotypic confirmatory tests for ESBL production were conducted by combined disk method using a third generation cephalosporins alone (cefotaxime, ceftriaxone, and ceftazidime) and in combination with clavulanic acid. A ≥ 5 mm in zone diameter for either of antimicrobial agent tested in combination with clavulanate vs the zone diameter of the agent when tested alone was considered positive for ESBL production based on CLSI guidelines [22].

### Carbapenemase production test

Phenotypic confirmatory tests for the ability of carbapenem-resistant isolates to produce Carbapenemase were investigated by the modified carbapenem inactivation method (MCIM) technique. A 1-μL loopful of the isolate to be tested was emulsified in 2ml trypticase soya broth (TSB) each and a 10-μg meropenem disk was immersed in each tube. After incubating for 4 hours ± 15 minutes the meropenem disk in the suspension was removed from each TSB-meropenem disk suspension and transferred to Mueller Hinton agar (MHA) plate already inoculated with *E*. *coli* ATCC 25922. The plate was then incubated at 35°c ± 2°c for 18 hours. Zone of inhibition of 6–15 mm and presence of pinpoint colonies within 16–18 mm zone were considered positive for carbapenemase production, according to CLSI guidelines [22].

### Multidrug resistance bacteria

Based on CLSI guidelines we have considered *E*. *coli* O157:H7 isolates multidrug resistant (MDR) that acquired non-susceptibility to more than one class of antimicrobials.

### Quality assurance

Prior to the data gathering procedure, stringent safeguards were put in place to assure data quality. WHO standardized data collection materials were used, which aided in the study’s consistency and homogeneity. Furthermore, after each phase of preparation, the sterility of the media used for sample inoculation was extensively verified by incubating it overnight. The integrity of the samples and subsequent analyses were maintained by guaranteeing the sterility of the medium, reducing the likelihood of false-positive outcomes. A positive American Type Culture Collection (ATCC) was used to validate the functionality of each culture medium used. In addition, at each phase of confirmatory test for ESBL and carbapenemases, both positive and negative controls were included [Positive control (*K*. *pneumoniae* ATCC 700603 for ESBL) and (*K*.*pneumoniae ATCC 1705* for Carbapenemase) and *E*. *coli* ATCC 25922 (negative control for both)].

### Data management and statistical analysis

The data were coded and entered to SPSS version 27.0 software for analysis. Categorical covariates were summarized using frequencies and percentages, and numerical variables were summarized with a mean or median value based on the data distribution symmetry. Bi-variant analyses were employed using a logistic regression model and multi-variant analyses were employed using a logistic regression model for variables that have *p*-value ≤0.25in their bivariant analyses and further interpreted based on the odds ratio and level of statistical significance at a *p*-value <0.05 with 95% confidence interval.

### Ethical approval and consideration

This study was approved by Departmental Research Ethics Review Committee (DRERC) of the Department of Microbiology, Immunology & Parasitology (DMIP), College of health science, Addis Ababa University (EC approval document number DRERC/002/2022), AHRI/ALERT Ethics Review Committee of Armauer Hansen Research Institute under Ethiopia Cholera Control and Prevention (ECCP) project (EC approval document number PO/11/21), and National Research Ethics Review Committee Ethiopia Cholera Control and Prevention (ECCP) project (EC approval document number 7/2-512/00259/35). In addition, before enrolling in the study, each study participant provided informed written consent/assent in accordance with established ethical procedures. During data collection and analysis, anonymity was maintained.

## Result

### Socio-demographic characteristics of the study participants

The median age of the study participants was 18 years with range of 1 to 75 years. Eighty-one (19.1%) of the participants were less than five years old and 14 (3.3%) of patients were elders aged above 55 years. The majority of the participants [262 (61.9%)] were males with a 1.6:1 male: female ratio.

Out of the 423 study participants, 258 (51.5%) lived in an urban area and more than half of the participants or guardians (in the case of children) [233 (55.1%)] attended only a primary school; and 126 (29.8%) study participants are farmers. In addition, the overwhelming majority of the participants [345 (81.6%)] had domestic animals. Tap water was the major source of drinking water for more than half of the participants [287 (67.8%)]. The majority [291 (68.7%)] of the participants had a habit of eating uncooked food (Table 1).

**Table 1:** Socio-demographic characteristics, magnitude, and associated factors of E. coli O157:H7 related diarrhea.

Variable	Category	Freq. N (%)	*E*. *coli* O157:H7 Positive. N (%)	Bivariant		Multivariant	
				P-value	COR (95%CI)	P-value	AOR (95%CI)
**Age group (Year)**	< = 5	81 (19.1%)	13 (34.2%)	1			
	6–15	102 (24.1%)	9 (23.7%)	0.0141	0.506 (0.205, 1.021)	0.313	0.497 (0.128, 1.930)
	16–25	133 (31.4%)	10 (26.3%)	0.056	0.425 (0.177, 1.021)	0.111	0.387 (0.121, 1.243)
	26–35	60 (14.2%)	4 (10.5%)	0.101	0.374 (0.115, 1.210)	0.156	0.339 (0.076, 1.510)
	36–45	25 (5.9%)	1 (2.6%)	0.152	0.218 (0.027, 1.756)	0.372	0.353 (0.036, 3.463)
	46–55	8 (1.9%)	1 (2.6%)	0.793	0.747 (0.085, 6.595)	0.858	0.808 (0.079, 8.267)
	> = 56	14 (3.3%)	0 (0%)	0.999	0		
**Residence**	Urban	218 (51.5%)	28 (73.7%)	1			
	Rural	205 (48.5%)	10 (26.3%)	0.006	0.348 (0.165, 0.736)	<0.001^a^	0.135 (0.050, 0.361)
**Educational status**	None	122 (28.8%)	14 (36.8%)	1			
	Primary	233 (55.1%)	20 (52.6%)	0.381	0.724 (0.352, 1.490)	0.305	1.780 (0.592, 5.353)
	Secondary	47 (11.1)	1 (2.6%)	0.089	0.168 (0.021, 1.313)	0.433	0.400 (0.040, 3.950)
	Collage	15 (3.5%)	2 (5.3%)	0.833	1.187 (0.242, 5.816)	0.252	3.184 (0.439, 23.105)
	University	6 (1.4%)	1 (2.6%)	0.702	1.543 (0.168, 14.178)	0.318	3.96 (0.267, 58.271)
**Family Size**	≤ 4	185 (43.7%)	24 (63.2%)	1			
	≥ 5	238 (56.3%)	14 (36.8%)	0.013	0.419 (0.210, 0.836)	0.002 ^a^	0.275 (0.120, 0.631)
**Domestic animal ownership**	No	78 (18.4%)	2 (5.3%)	1			
	Yes	345 (81.6%)	36 (94.7%)	0.044	4.427 (1.043, 18.795)	0.013 ^a^	7.154 (1.510, 33.890)
**Source of drinking water**	Tap	297 (70%)	19 (50%)	1			
	Spring	31 (7.3%)	1 (2.6%)	0.492	0.488 (0.063, 3.773)	0.828	1.283 (0.136, 12.122)
	Well	72 (17%)	16 (42.1%)	<0.001	4.180 (2.026, 8.626)	0.004 ^a^	3.639 (1.506, 8.792)
	River	23 (5.4%)	2 (5.3%)	0.669	1.393 (0.304, 6.391)	0.167	3.508 (0.592, 20.771)
**Drinking raw milk**	No	214 (50.6%)	24 (63.2%)	1			
	Yes	209 (49.4%)	14 (36.8%)	0.108	0.568 (0.285, 1.132)	0.604	0.814 (0.374, 1.772)
**Blood in diarrhea** *	No	408 (96.5%)	32 (84.2%)	1			
	Yes	15 (3.5%)	6 (15.8%)	<0.001	7.833 (2.623, 23.397)	<0.001 ^a^	12.465 (2.824, 55.018)
**Abdominal Cramp** *	No	323 (76.4%)	24 (63,2%)	1			
	Yes	100 (23.6%)	14 (36,8%0	0.048	2.028 (1.006, 4.090)	0.337	1.626 (0.603, 4.385)

Key Notes: AOR, adjusted odds ratio; COR, crude odds ratio; CI, confidence interval; 1, Reference;

*, clinical condition; (Others: -Prisoners, commercial sex workers, laborers, housewives, and no job, ^a^ statistically significant).

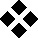
 Only those variables with P-value of ≤0.25 on their Bivariant analysis is incorporated in the table.

#### Magnitude and associated factors of *E*. *coli* O157:H7-related diarrhea

All the 423 participants selected for the study were presented with watery diarrhea that lasted several hours or days, of whom 13.2% had mucus and 3.5% had dysentery. Upon laboratory investigation, we isolated *E*. *coli* isolates from 396/423 (93.6%) of diarrheal sample. *E*. *coli* O157:H7 strain was found in 38 (9%) of the study participants. Living in an urban area (AOR: 0.135; 95% CI: 0.050, 0.361), having a domestic animal (AOR: 7.154; 95% CI: 1.510, 33.890), and having ≥5 family size (AOR: 0.275; 95% CI: 0.120, 0.631) were significantly associated with *E*. *coli* O157:H7 cases (Table 1).

#### Magnitude of ESBL and carbapenemase producing *E*. *coli* O157:H7

From the total of 38 *E*. *coli* o157:H7 isolates, 27 (71.1%) and 12 (31.6%) were phenotypically confirmed ESBL and carbapenemase producers, respectively. All the 27 study participants with ESBL-producing isolates owned domestic animals and the source of drinking water for the majority of study participants with ESBL-producing isolates 14 (52%) was well water (Table 2).

**Table 2:** Magnitude of ESBL and carbapenemase producing *E*. *coli* O157:H7 and its distribution among different socio-demographic status of the study participants.

Variables	Category	ESBL Positive (N = 27)	P-value	COR (95%CI)	Carbapenemase Positive (N = 12)	P-value	COR (95%CI)
**Residence**	Urban	19 (70%)	0.86	1.442(0.026, 78962)	10 (83%)	0.47	3.14 (0.138, 71.56)
	Rural	8 (30%)			2 (17%)		
**Gender**	Male	17 (63%)	0.6	0.422 (0.018, 9.872)	8 (67%)	0.82	1.29 (0.143, 11.62)
	Female	10 (37%)		-	4 (33%)		-
**Occupation**	None	8 (30%)	0.99	-	4 (33%)	0.994	-
	Student	9 (33%)	0.99	-	4 (33%)	0.994	-
	Farmer	7 (26%)	0.99	-	2 (17%)	0.994	-
	Merchant	2 (7%)	-	-	0 (0%)		-
	Employee	0 (0%)	-	-	0 (0%)		-
	Others	1 (4%)	-	-	2 (17%)		-
**Domestic animal ownership**	No	0 (0%)	-	-	1 (8%)	0.998	-
	Yes	27(100%)	-	-	11 (92%)		-
**Source of drinking water**	Tap	11 (41%)	0.99	-	4 (33%)	0.226	0.057 (0.001, 5.908)
	Spring	1 (4%)	0.99	-	1 (8%)	0.998	-
	Well	14 (52%)	0.99	-	6 (50%)	0.465	0.201 (0.003, 14.83)
	River	1 (4%)	-	-	1 (8%)		-
**Drinking raw milk**	No	18 (67%)		-	7 (58%)	0.405	0.288 (0.015, 5.38)
	Yes	9(33%)	0.21	17.34(0.194,1554.9)	5 (42%)		-
**Eating uncooked food**	No	7(26%)	0.99	-	2 (17%)	0.939	0.871 (0.025, 30.41)
	Yes	20(74%)		-	10 (83%)		-

#### Overall antimicrobial resistance status of *E*. *coli* O157:H7 isolates

**Fig 1:**
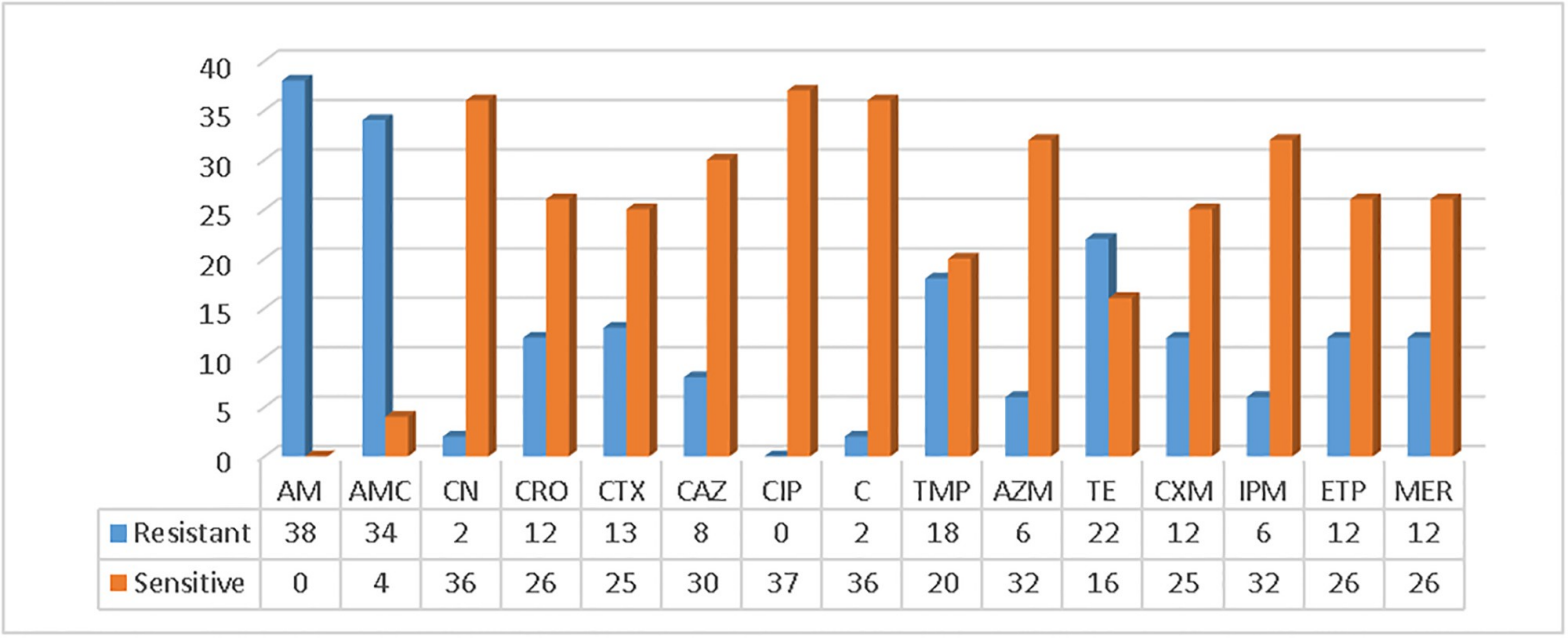
Antimicrobial resistance status of *E*. *coli* O157:H7 isolates. **Keynote:-** AM = Ampicillin, AMC = Amoxicillin/Clavulanic acid, CN = Gentamycin, CRO = Ceftriaxone, CTX = Cefotaxime, CAZ = Ceftazidime, CIP = Ciprofloxacin, C = Chloramphenicol, TMP = Trimethoprim/sulfamethoxazole, AZM = Azithromycin, TE = Tetracycline, CXM = Cefuroxime, IPM = Imipenem, ETP = Ertapenem, MER = Meropenem.

The antimicrobial susceptibility of the 38 isolates was evaluated against 15 antimicrobial agents by the Kirby Bauer disk diffusion method. High antimicrobial resistance was observed against Ampicillin [38 (100%)], Amoxicillin with clavulanic acid [34 (89.5%)], tetracycline [22 (57.9%)], Ertapenem [12 (31.6%)], and Meropenem [12 (31.6%)] in reverse, all isolates were sensitive to ciprofloxacin. (Fig 1).

#### Antimicrobial resistance status of ESBL and carbapenemase-producing *E*. *coli* O157:H7 isolates

**Fig 2:**
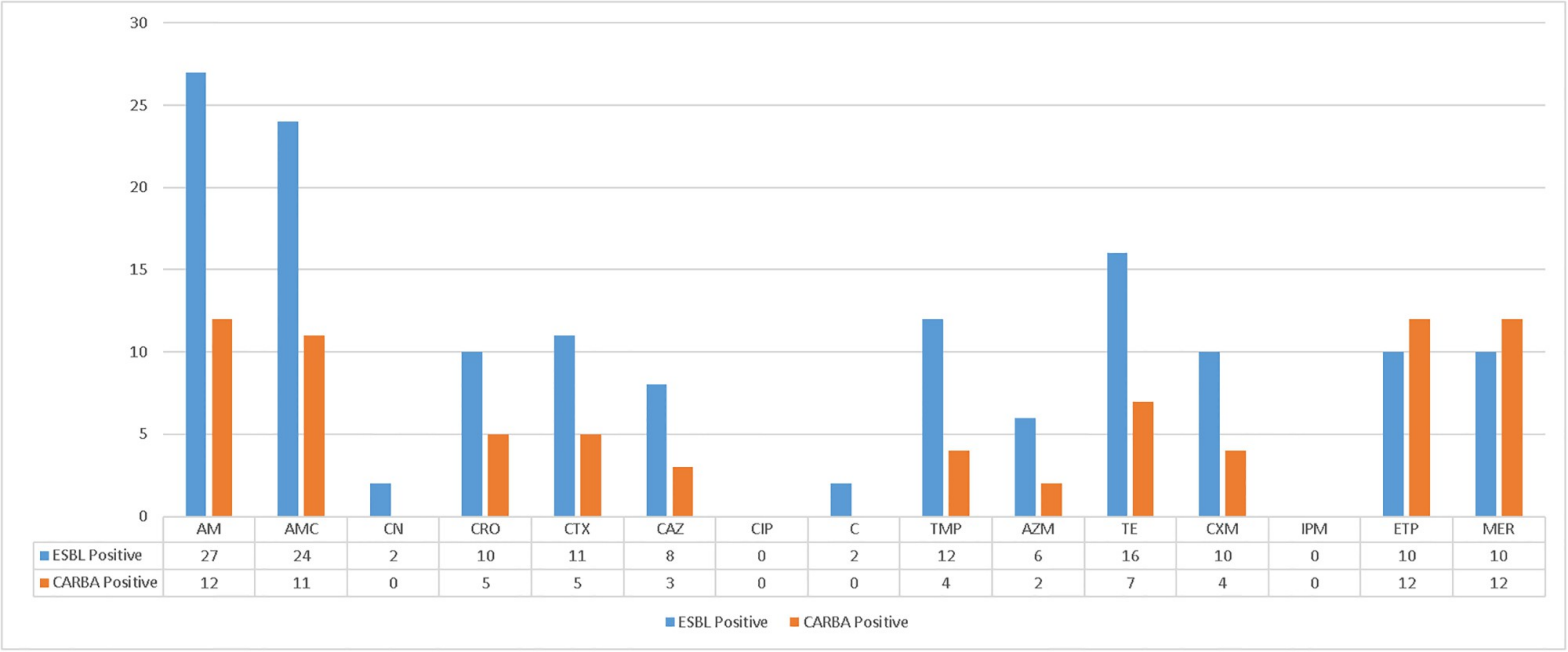
Antimicrobial resistance status of ESBL and carbapenemase-producing isolates. **Keynote**:—AM = Ampicillin, AMC = Amoxicillin/Clavulanic acid, CN = Gentamycin, CRO = Ceftriaxone, CTX = Cefotaxime, CAZ = Ceftazidime, CIP = Ciprofloxacin, C = Chloramphenicol, TMP = Trimethoprim/sulfamethoxazole, AZM = Azithromycin, TE = Tetracycline, CXM = Cefuroxime, IPM = Imipenem, ETP = Ertapenem, MER = Meropenem.

All ESBL and carbapenemase-producing *E*. *coli O157*:*H7* isolates were resistant to ampicillin but sensitive to ciprofloxacin and imipenem. Varied levels of resistance were observed among these groups against the tested antimicrobials between these extremes, ranging from 16/27 (59.25%) against Tetracycline to 2/27 (7.41%) against chloramphenicol and Gentamycin by ESBL-positive isolates; and from 11/12 (91.70%) against Amoxicillin/Clavulanic acid to 2/12 (16.70%) against Azithromycin by Carbapenemase-positive isolates (Fig 2).

### Magnitude of multi-drug resistant (MDR) isolates

**Fig 3:**
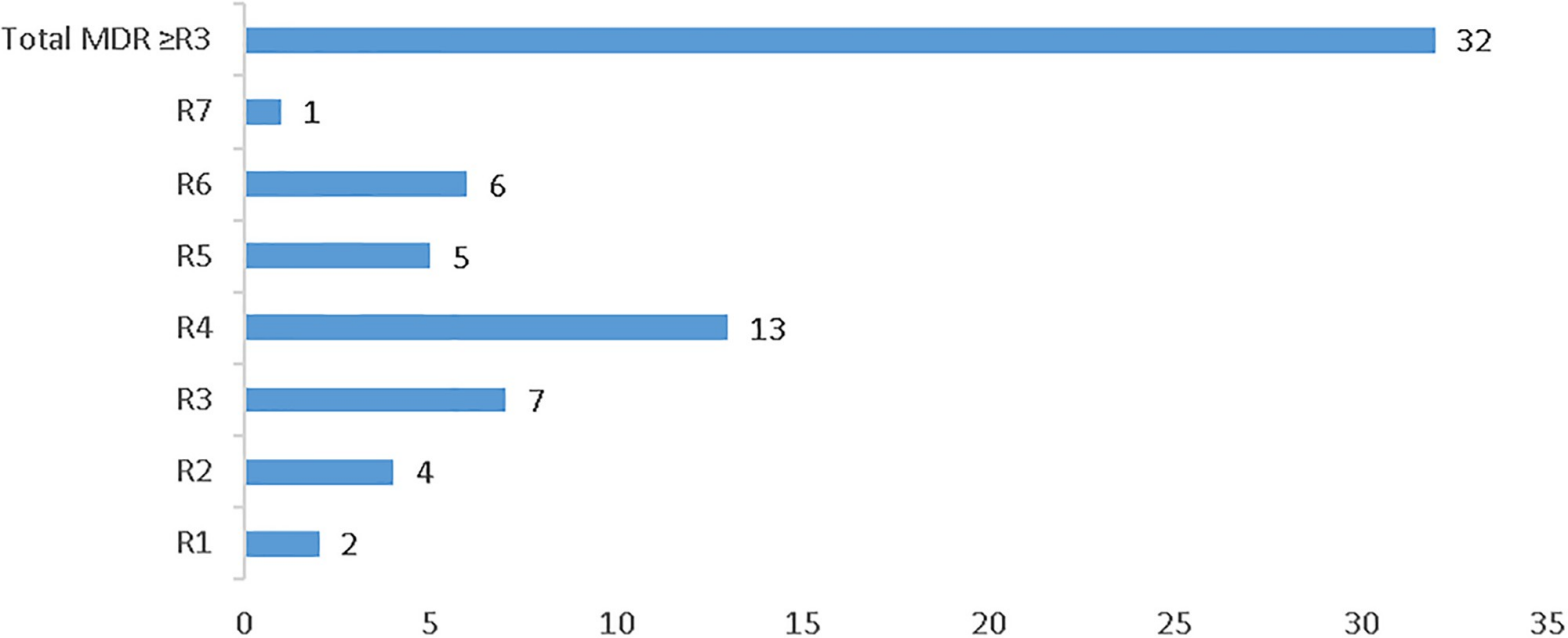
Magnitude of multi-drug resistant isolates. **Keynote:-** R1 = Resistance to one class of antimicrobial, R2 = Resistance to two class of antimicrobial, R3 = Resistance to three class of antimicrobial, R4 = Resistance to four class of antimicrobial, R5 = Resistance to five class of antimicrobial, R6 = Resistance to six class of antimicrobial, R7 = Resistance to seven class of antimicrobial, MDR = resistance to ≥3 class of antimicrobial.

Resistance to three or more classes of antimicrobial is recorded on 32/38 (84.2%) isolates. Among these, 13 (34.2%) isolates were resistant to 4 classes of antimicrobials while only 1 (2.6%) isolate was resistant to 7 classes of antimicrobials (Fig 3).

## Discussion

In our study the presence of *E*. *coli* O157:H7 is found to be 9% of diarrheal cases. The isolated bacteria displayed resistance to various antimicrobial agents, but showed susceptibility to ciprofloxacin. In addition to that, phenotypic confirmation revealed that 71.1% of the bacterial isolates were ESBL producers, while 31.6% were carbapenemase producers and 26.3% isolates tend to produce both ESB and carbapenemase. All ESBL-producing and 92% carbapenemase producing isolates come from participants that own domestic animals but does not have significant association.

Our finding shows a slightly higher magnitude of *E*. *coli* O157:H7 than the study from Wolaita Sodo [6.03%; [23]], and Bahirdar [6.8%; [24]] both of which were conducted on under-five children. However, it is significantly higher than the report from Addis Ababa [4.5%; [14]] and Bishoftu town [2.8%; [25]]. Conversely, it is lower than the rate reported from Eastern Ethiopia[15.3%; [26]]; and much lower than rates detected from studies conducted in other Africa countries (Gabon (63.5%) and Nigeria (44.5%)) [27, 28]. These differences may be due to cultural, lifestyle, and sanitation status disparities among the study populations.

Our study also found that both adults and under-five children living in urban areas are susceptible to *E*. *coli* O157:H7 infection compared to those residing in rural areas, a finding which is in disagreement with another study conducted in eastern Ethiopia [26] which shows children living in a rural area were prone to *E*. *coli* O157:H7 infection than those living in an urban environments. The disparity could be attributed to factors such as higher population density, inadequate sanitation, reliance on food distribution networks, limited access to safe water, presence of animals, and urban lifestyle practices [29–31].

The prevalence of ESBL production is somewhat comparable to the findings from Egypt [63%, [19]] but much higher than the findings from Tanzania [9.3%, [20]]. The difference in the distribution of ESBL producers could be because of variations in geographic location, study population, and differences in antibiotic prescribing practices and usage patterns between the research area in this study and the aforementioned locations. Different classes of antibiotics exert selective pressure, which can promote the establishment and spread of various ESBL genes. The use of antibiotics, including different types, dosages, and durations, can contribute to changes in the prevalence and distribution of ESBL genes in a specific area [13, 32]. As for the distribution of carbapenemase producer’s since, this is the first study conducted to detect the carbapenemase production in *E*. *coli* O157:H7, we were unable to find any other studies to compare with our findings.

The *E*. *coli* O157:H7 isolated in our study demonstrated significant antimicrobial resistance to Ampicillin [38/38 (100%)], Amoxicillin-clavulanic acid [34/38 (89.5%)], Tetracycline [22/38 (57.9%)], and an equal rate of 12/38 (31.6%) resistance to Ertapenem and Meropenem. However, all isolates exhibited sensitivity to ciprofloxacin. These findings align with those of previous studies conducted in Bahirdar [24], Ambo Town, [6], Jimma [33], Addis Ababa [34], and central Ethiopia [35], which reported high resistance rates to ampicillin and susceptibility rates above 93% to ciprofloxacin. Furthermore, 32 (84.2%) of the isolates demonstrated resistance to three or more classes of antimicrobials, consistent with the study conducted in Bahirdar which reported around 88% multidrug resistance. This high level of multidrug resistance raises concerns about therapeutic options, potentially resulting in treatment failures and increased morbidity/mortality rates.

## Conclusions

During our laboratory investigation in Shashemene town and its surrounding areas, we found that 9% of diarrheal patients were infected with *E*. *coli* O157:H7 bacteria. Among these isolates, a high percentage (71.1%) produced ESBL, and 31.6% produced Carbapenemase. Fortunately, all isolates were susceptible to the antibiotic Ciprofloxacin, making it the preferred treatment option. However, in addition to ESBL and carbapenemase production, we observed an alarmingly high MDR rate of 84% among the *E*. *coli* O157:H7 strains in our study. These findings calls for a thorough epidemiological investigation on antimicrobial resistance that incorporates clinical, environmental and animal data across various sectors. By adopting such an approach, we can gain a deeper understanding of E. coli O157infections and antimicrobial resistance, especially concerning the sources of infection and their clinical consequences.

## Supporting information

S1 File Shimelis Teshome data *E*. *coli* O157. (XLSX)
